# Metastability for discontinuous dynamical systems under Lévy noise: Case study on Amazonian Vegetation

**DOI:** 10.1038/s41598-017-07686-8

**Published:** 2017-08-24

**Authors:** Larissa Serdukova, Yayun Zheng, Jinqiao Duan, Jürgen Kurths

**Affiliations:** 1 School of Mathematics and Statistics, Huazhong University of Science and Technology, Wuhan, 430074 China; 2 Center for Mathematical Sciences, Huazhong University of Science and Technology, Wuhan, 430074 China; 3 Department of Science and Technology, University of Cape Verde, Praia, 7600 Cape Verde; 4 Wuhan National Laboratory for Optoelectronics, Wuhan, 430074 China; 5 Department of Applied Mathematics, Illinois Institute of Technology, 312-567-5335, Chicago, 60616 USA; 6 Research Domain on Transdisciplinary Concepts and Methods, Potsdam Institute for Climate Impact Research, PO Box 60 12 03, 14412 Potsdam, Germany; 7 Department of Physics, Humboldt University of Berlin, Newtonstrate 15, 12489 Berlin, Germany

## Abstract

For the tipping elements in the Earth’s climate system, the most important issue to address is how stable is the desirable state against random perturbations. Extreme biotic and climatic events pose severe hazards to tropical rainforests. Their local effects are extremely stochastic and difficult to measure. Moreover, the direction and intensity of the response of forest trees to such perturbations are unknown, especially given the lack of efficient dynamical vegetation models to evaluate forest tree cover changes over time. In this study, we consider randomness in the mathematical modelling of forest trees by incorporating uncertainty through a stochastic differential equation. According to field-based evidence, the interactions between fires and droughts are a more direct mechanism that may describe sudden forest degradation in the south-eastern Amazon. In modeling the Amazonian vegetation system, we include symmetric *α*-stable Lévy perturbations. We report results of stability analysis of the metastable fertile forest state. We conclude that even a very slight threat to the forest state stability represents L´evy noise with large jumps of low intensity, that can be interpreted as a fire occurring in a non-drought year. During years of severe drought, high-intensity fires significantly accelerate the transition between a forest and savanna state.

## Introduction

Tipping elements (TEs) are subsystems of the Earth’s climate system, at least subcontinental in scale, which are characterized by a critical control value, called the tipping point, beyond which even small perturbations of the system may lead to drastic qualitative changes in the system’s features and behaviour^1^. The study of these systems plays a crucial role in interdisciplinary research, particularly because TEs represent a significant part of our planet. The smooth functioning of TEs directly depends on its performance and may also have a significant impact on humans and their welfare. Thus, it has now become a challenge to quantify the qualitative changes in TEs in terms of the impact that they might have on all elements of an ecosystem.

One such tipping elements is the Amazon rainforest. In recent years, the rainforest has attracted substantial attention from scientists from different areas. Menck *et al*. have proposed a conceptual model that describes the dynamical behaviour of the forest cover^2–4^ incorporating non-smooth switches in the growth term. Sternberg *et al*. have developed an accurate forest area model that addresses forest contributions to dry season precipitation and the consequential effects on the forest’s own establishment^3^. Hirota *et al*. have analysed data on the distribution of tree cover in Africa, Australia and South America, and have statistically tested their hypothesis describing the existence of three distinct attractors in a forest ecosystem: forest, savanna and treeless states^4^.

However, in the modelling of the Amazon rainforest, there is only a small amount of information^5, 6^ on an issue that deserves attention, given its importance: How robust are desirable, i.e. present, fertile forest states against random and even large perturbations^2^. Global warming-based, droughts, wildfires and similar biotic events pose severe hazards to the tropical forest. However, the direction and intensity of the rainforest’s vegetative response to extreme climatic events are uncertain, given the lack of efficient vegetation models to evaluate changes in forest tree cover under climatic influence. Some attempts to measure the highly stochastic effects of extreme events on forest ecosystems have already been made; e.g., Manso *et al*.^7^ have presented an empirical single-tree mortality model for multi-species stands that considers competition and extreme event-mediated mortality. The latter is included in the model via random effects that considers the stochastic nature of the phenomenon. A coupled approach combining dendrochronology and ecophysiology has been used by Bréda *et al*.^8^ to illustrate how some extreme events affect forest ecosystems and to provide various management guidance in order to moderate extreme drought and control selective mortality. Another attempt to clarify this subject has been made by Rammig *et al*.^9^ and consists in estimating the risk of Amazonian forest dieback by using weighted rainfall projections from general circulation models to create probability density functions of future forest biomass changes. Additionally, Zeng *et al*.^10^ have developed an empirical approach, based on the observed climatic spacing of tropical trees, to estimate the maximum potential tropical tree cover with a given climate. Their results emphasize the importance of a temperature, precipitation, and atmospheric *CO*_2_ in determining tropical tree coverage.

Current approaches used in modelling forest response to extreme events, both mechanistic and empirical, have limitations. In particular, they require very precise data, which are not always available, as well as data from damage produced by a limited number of extreme events, thus resulting in significant biases in the predictions extrapolated by these models. Models from catastrophe theory with bifurcation points proposed for the switch between forest cover and the alternative stable state of grassland are deterministic systems, even though the deterministic nature of these systems does not make them predictable, their future behavior is fully determined by their parameters and initial conditions, with no random elements involved. However, these extreme events and their local effects, are extremely stochastic in nature and are difficult to measure.

Given this background, it is important to consider randomness in the mathematical modelling of forest tree cover with uncertainty and to devise a stochastic differential equation for the evolution of forest ecosystems, as influenced by extreme climatic and biotic events. Moreover, field-based evidence suggests that the response of forest cover to drought-fire interactions will not be smooth but will exhibit sudden transitions^11^. These abrupt increases in fire-induced tree mortality (226 and 462 percent) have been found during the grim drought season, on the basis of results of a large-scale, long-term experiment with annual and triennial burn regimes. A Lévy process with jumps^12, 13^ is the best choice among the stochastic processes to model such abrupt pulses given their special properties such as heavy-tailed distributions and stochastically continuous sample paths. Therefore, we will includ random perturbations of symmetric *α*-stable Lévy type in the deterministic conceptual Amazonian vegetation model and perform a stability analysis of the metastable fertile forest state. In fact, the resulting stochastic model demonstrates the dynamical behaviour called metastability, in which a system explores the state space on different time scales: the fast time scale is the transitions that occurs within a single subregion, and the slow time scale occurs between different subregions^14^. Analysing a data set of forest cover to demonstrate Lévy flight behaviour, as well as performing a power-law test on Lévy distributions in forest ecosystems^15^ was outside the scope of our study. To our knowledge our research is the first attempt to model forest cover by taking the stochastic nature of forest ecosystems into consideration.

The remainder of the paper is organized as follows. In **Model and Methods**, we describe the deterministic conceptual Amazonian vegetation model. It includes a particular difficulty of a discontinuous vector field, which is a known non-smooth dynamical system model. In this section, we raise questions such as the notion of the existence and uniqueness of solutions, and we show that our system holds the repulsive sliding mode vector field around the switching boundary *x* = *X*_*crit*_, thus suggesting that the initial value problem, with the initial condition *x*_0_ = *X*_*crit*_ has three possible solutions. We propose a smooth approximation of the discontinuous vector field performed by using a mollification method to overcome the problem of non-uniqueness. The perturbation type, its main characteristics and the stochastic Amazonian vegetation system are also discussed in this section. In **Results and Discussion**, we carry out the stability analysis via a first exit time, an escape probability and a stochastic basin of attraction. The results obtained are also presented. Finally, we summarize our findings in the **Conclusion**.

## Model

### Conceptual Amazonian vegetation model (AV)

The growth dynamics of the forest cover in Amazonian rainforests have been described by Menck *et al*.^2^ via the Levins model^16, 17^, in which a non-smooth switch in the growth term that represents the tipping point was incorporated:

1 $$\frac{dx}{dt}=f(x)=\{\begin{array}{cc}G(1-x)x-Dx & {\rm{i}}{\rm{f}}\,x > {X}_{crit},\\ -Dx & {\rm{i}}{\rm{f}}\,x < {X}_{crit},\end{array}$$

where *x* is the relative forest cover that grows with the saturating rate *G* if *x* > *X*_*crit*_ and dies with rate *D* (assuming *G* > *D* > 0). This model has two equilibria: the forest state ${x}_{F}=1-\frac{D}{G}$ and the savanna state *x*_*S*_ = **0**. *x*_*F*_ (resp. *x*_*S*_) exists and is stable if *x*_*F*_ > *X*_*crit*_ (resp. *X*_*crit*_ > 0). *X*_*crit*_ is the critical forest cover threshold and directly depends on aridity. Owing to global warming, aridity has a tendency to increase, thus causing a significant displacement of *X*_*crit*_ and consequently a size decrease in the basin of the forest state, thus contributing to the *x*_*F*_ instability before the perturbations. When *X*_*crit*_ reaches the level of *x*_*F*_, the forest state disappears.

In the mathematical literature, this type of dynamical system is known as piecewise-smooth dynamics^18, 19^ or non-smooth dynamical systems, which are described by differential equations with a discontinuous right-hand side^20, 21^. Numerous fundamental questions arise when working with discontinuous dynamical systems. The most basic question is the notion of a solution. Many researchers have contributed to the foundation of this issue^22^, including Filippov, Caratheodory, Krasovskii, Euler and Hermes. However, for our purpose we chose the generalized definition of the solution based on the Filippov theory^21^. Therefore, the AV model can be described by a more general *n*-dimensional nonlinear system with a discontinuous right-hand side^20^:

2 $$\dot{x}(t)=f(t,x(t))=\{\begin{array}{cc}{f}_{+}(t,x(t)), & x\in {{\mathcal{V}}}_{+},\\ {f}_{-}(t,x(t)), & x\in {{\mathcal{V}}}_{-},\end{array}$$

where the initial condition *x*(0) = *x*_0_. The right-hand side *f*(*t*, *x*) is assumed to be piecewise-continuous and smooth on ${{\mathcal{V}}}_{-}$ and ${{\mathcal{V}}}_{+}$ and discontinuous on the hyper-surface Σ, which is called the *switching boundary*. The boundary is defined by a scalar switching boundary function *h*(*x*). In our model Σ = {*x* = *X*_*crit*_}; thus, *h*(*x*) = *X*_*crit*_ − *x*. The function *f*_−_(*t*, *x*) is therefore assumed to be *C* ^1^ on ${{\mathcal{V}}}_{-}\cup {\rm{\Sigma }}$, and *f*_+_(*t*, *x*) is assumed to be *C* ^1^ on ${{\mathcal{V}}}_{+}\cup {\rm{\Sigma }}$. These functions do not agree at the boundary Σ. The system described by (2) does not define *f*(*t*, *x*(*t*)) if *x*(*t*) is on Σ. One of the methods to overcome this problem is known as Filippov’s convex method^21^, which consists of an extension (or convexification) of (2) into the following convex differential inclusion (CDI) (or set-valued extension) *F*(*t*, *x*) is the general case and in our model:

3 $$\begin{array}{ccc}\dot{x}(t)\in F(t,x(t)) & = & \{\begin{array}{cc}{f}_{+}(t,x(t)), & x\in {{\mathcal{V}}}_{+},\\ \overline{co}\,[{f}_{-}(t,x(t)),{f}_{+}(t,x(t))], & x\in {\rm{\Sigma }},\\ {f}_{-}(t,x(t)), & x\in {{\mathcal{V}}}_{-},\end{array}\\  & = & \{\begin{array}{cc}G(1-x)x-Dx, & x > {X}_{crit}\\ \overline{co}\,[G(1-x)x-Dx,-Dx], & x={X}_{crit},\\ -Dx, & x < {X}_{crit},\end{array}\end{array}$$

where $\overline{co}\,[{f}_{-},{f}_{+}]$, the convex set with two right-hand side *f*_−_ and *f*_+_, is represented by the following equation:

4 $$\begin{array}{rcl}\overline{co}\,[{f}_{-},{f}_{+}] & = & [(1-q){f}_{-}+q{f}_{+},\,\forall q\in [0,1]]\\  & = & [qG\mathrm{(1}-x)x-Dx,\,\forall q\in [0,1]\,]\\  & = & [-D{X}_{crit},\,G\mathrm{(1}-{X}_{crit}){X}_{crit}-D{X}_{crit}].\end{array}$$

The solution concept in the sense of the Filippov method^20^ (definition 3.3, page 32) guarantees the existence of a solution for the system (3) under the assumption that the set-valued function *F*(*t*, *x*) is upper semi-continuous. The notion of upper semi-continuity can be found in ref. 20 chapter 2, section 2.2, and the existence of a solution of a differential inclusion theorem with a proof is given in ref. 23 (Theorem 3, page 98).

The second fundamental question for this piecewise-smooth dynamical system is the uniqueness of the solution. Obviously, the solution of the initial valued problem (IVP) equation (3) where ${x}_{0}\notin {\rm{\Sigma }}$ is locally unique because *f*_−_(*t*, *x*) and *f*_+_(*t*, *x*) are smooth. Uniqueness problems of IVP may arise when *x*_0_ ∈ Σ or the solution crosses the switching boundary Σ. The solution of the differential inclusion (3) with *x*_0_ ∈ Σ does not satisfy the local uniqueness condition in forward time, as presented in the **Methods** section. In fact, the type of vector field, defined by (3), is around the switching boundary *x* = *X*_*crit*_ and is called a *repulsive* (*repelling*) *sliding mode* ^18^ after the solutions diverge from *x* = *X*_*crit*_, i.e., a solution that starts close to *x* = *X*_*crit*_ will move away from it. However, a solution with the initial condition *x*_0_ = *X*_*crit*_ may stay on *X*_*crit*_ (because 0 ∈ *F*(*X*_*crit*_)) or leave the switching boundary by entering either ${{\mathcal{V}}}_{-}$ or ${{\mathcal{V}}}_{+}$. The IVP with the initial condition *x*_0_ = *X*_*crit*_ has three possible solutions:

5 $$x(t)=\{\begin{array}{c}-\frac{D-G}{2G}\cdot (\tanh (\frac{({C}_{1}-t)\,(D-G)}{2})+1),\\ {X}_{crit},\\ {X}_{crit}\cdot \exp \,(-Dt),\end{array}$$

where ${C}_{1}\in {{\mathbb{R}}}^{1}$. Varying the parameters *D*, *G* and *X*_*crit*_ (i.e. *G* > *D* > 0 and *x*_*F*_ > *X*_*crit*_) do not lead the system to a bifurcation. The system experiences structural instability only when *X*_*crit*_ reaches the *x*_*F*_ level. Thus, considering the objectives of our analysis, we assume that *D* = 0.2, *G* = 0.85 and *X*_*crit*_ = 0.3. In this case, the convex differential inclusion *F*(*t*, *x*) (3) of the discontinuous AV system (1) is as follows:

6 $$F(x)=\{\begin{array}{cc}-0.85{x}^{2}+0.65x, & {\rm{i}}{\rm{f}}\,x > \mathrm{0.3.}\\ \left[-0.06,0.1185\right], & {\rm{i}}{\rm{f}}\,x=\mathrm{0.3.}\\ -0.2x, & {\rm{i}}{\rm{f}}\,x < \mathrm{0.3.}\end{array}$$

**Figure 1:**
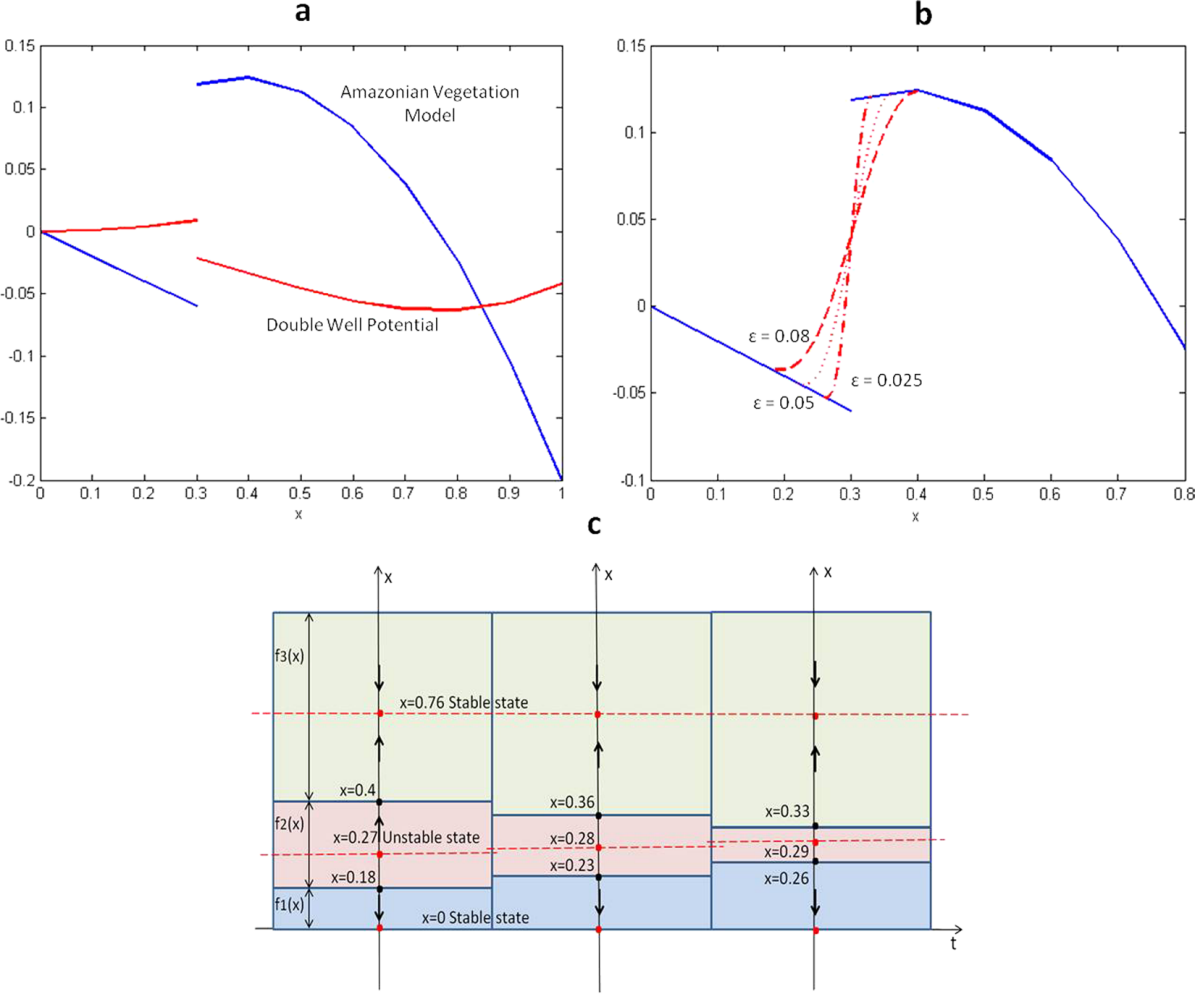
(**a**) Discontinuous vector field of Amazonian vegetation model (blue line) and respective double well potential (red line). (**b**) Phase portrait for mollified model with *ε* = 0.08, 0.05, 0.025. (**c**) Mollified vector field *f* ^*ε*^(*x*). Dashed line (*ε* = 0.08), dotted line (*ε* = 0.05) and dash-dotted line (*ε* = 0.025) with support in (0.177, 0.404), (0.227, 0.362) and (0.264, 0.330), respectively.

The discontinuous vector field of the Amazonian vegetation model is shown in Fig. 1(a).

### Smooth approximation

To overcome the problem of non-uniqueness of solutions in the non-smooth dynamical system, and to be able to apply the stability analysis techniques for the metastable states in the case of stochastically perturbed dynamical system, we will achieve a smooth approximation of the discontinuous vector fields. Often, smoothing methods are used to address complicated bifurcations^19^, difficulties in numerical integration^20, 24^ or problems with the existence and uniqueness of the solution, which arises in differential inclusions with sliding modes. However, this method has some disadvantages, including that it generates stiff differential equations which are numerically expensive to solve, and it does not always lead to the approximations that convey the physical reality^20^ in the best way. However, in our case, this approximation is useful, because the smoothness of the vector field is a necessary property in our analysis.

Among the wide variety of smooth approximation methods, the method that stands out is the mollification method. This method was chosen because of the large number of benefits that it provides during the regularization of many ill-posed problems^25–29^, including efficiency, accuracy, robustness, and reduced costs. A detailed explanation of how we carry-out the mollification of (3) is found in the **Methods** section. Our mollified AV model is now described by the following differential equation with a piecewise-smooth continuous vector field (all computations were performed in Matlab):

7 $$\frac{dx}{dt}={f}^{\varepsilon }(x)=\{\begin{array}{cc}-0.85{x}^{2}+0.65x, & {\rm{i}}{\rm{f}}\,x > {U}_{\varepsilon }^{h},\\ {A}^{\ast }, & {\rm{i}}{\rm{f}}\,{U}_{\varepsilon }^{l}\le x\le {U}_{\varepsilon }^{h},\\ -0.2x, & {\rm{i}}{\rm{f}}\,x < {U}_{\varepsilon }^{l},\end{array}$$

where ${U}_{\varepsilon }=[{U}_{\varepsilon }^{l},\,{U}_{\varepsilon }^{h}]$, *U* = [*U* ^*l*^, *U* ^*h*^] and *A* ^*^ is represented by:

8 $$\begin{array}{rcl}{A}^{\ast } & = & {\eta }_{\varepsilon }\ast f\\  & = & {\int }_{{U}^{l}}^{0.3}\,(-0.2t\cdot \frac{1}{\varepsilon \sqrt{\pi }}\,\exp \,(-\frac{{(x-t)}^{2}}{{\varepsilon }^{2}}))\,dt\\  &  & +{\int }_{0.3}^{{U}^{h}}\,(-0.85{t}^{2}+0.65t)\cdot \frac{1}{\varepsilon \sqrt{\pi }}\,\exp \,(-\frac{{(x-t)}^{2}}{{\varepsilon }^{2}}))\,dt.\end{array}$$

In this analysis, we consider three distinct values for the parameter *ε*, obtaining the following intervals for *U* and *U*_*ε*_:

i) for *ε* = 0.08 *U*_*ε*_ = [0.177, 0.404] and *U* = [0.100, 0.495];
ii) for *ε* = 0.05 *U*_*ε*_ = [0.227, 0.362] and *U* = [0.170, 0.420];
iii) for *ε* = 0.025 *U*_*ε*_ = [0.264, 0.330] and *U* = [0.235, 0.360];

The mollification of the original AV model changes the dynamical behaviour of the system in the neighbourhood of the discontinuity *x* = *X*_*crit*_ = 0.3, transforming the repulsive sliding mode in the unstable equilibria *x*_*A*_ = 0.27 (resp. 0.28, 0.29) for *ε* = 0.08 (resp. 0.05, 0.025). The vector field *f* ^*ε*^(*x*) and the phase portrait of the mollified AV model are shown in Fig. 1(c,b).

### Randomly perturbed Amazonian vegetation system

Previous research in environmental sciences^7, 11^ has provided field-based evidence for the premise that one of the important variables of a landscape, such as tree cover, experiences drastic variations or sharp transitions in responding to climate changes and other stressors. High-intensity fires associated with severe drought may accelerate a widespread degradation of Amazonian forests by abruptly increasing tree mortality^11^. When forest fires do occur under average weather conditions, they typically move slowly, liberating little energy, and they are short in duration and end at night when relative humidity increases. During years of severe drought, the fuel (e.g., leaves, twigs and branches) becomes more abundant and drier, thereby increasing the fires intensity and consequently killing a very high percentage of the trees. An extreme event acts either as an external disturbance, which forest systems can resist, or as a disturbance exceeding the resilience of forest ecosystems and preventing to return to the former dynamical state^8^, thus indicating the presence of the tipping point. Because the interactions between fires and droughts are a more direct mechanism of sudden forest degradation in the south-eastern Amazon^11^, the dynamical model of forest cover evolution goes beyond the typical tipping point, which is modelled via discontinuity in the vector fields; hence, the model must contain a stochastic term that fits perturbations such as fires. Recently, to model these abrupt pulses, burst-like or extreme events have been given higher priority to Lévy perturbations with jumps^12, 13, 30^, because of their properties, such as heavy-tailed distributions, and noncontinuous sample paths. The probabilistic description of the Lévy process is introduced in the **Methods** section. This description includes a more intuitive but concise premise of the concept that should be understandable to a broad audience. In our stochastically perturbed AV model we incorporate perturbations of only climatic nature, such as climatic changes^31^, aridity^2^, precipitations^3, 4^ and fires^11^, which the model may response to by exhibiting metastability. In this way, we consider the fourth case of perturbations, described in the **Methods** section, which includes the symmetric *α*-stable Lévy process. The time that the process spends below the value zero is regarded as the hibernation time^32^ (as exhibited by plants adapted to a desert environment), in which the trees that are more robust to the aridity can remain in dry state *x*_*S*_ = 0 (without dying) for a long time until rain comes.

Following the above discussion, we include random perturbations of ${L}_{t}^{\alpha }$ type in system (7) and obtain the following SDE in ${{\mathbb{R}}}^{1}$,

9 $$d{X}_{t}={f}^{\varepsilon }({X}_{t})dt+\psi d{L}_{t}^{\alpha },\,{X}_{0}=x,$$

where *f* ^*ε*^ is the mollified drift, and *ψ* is the noise intensity parameter.

**Figure 2:**
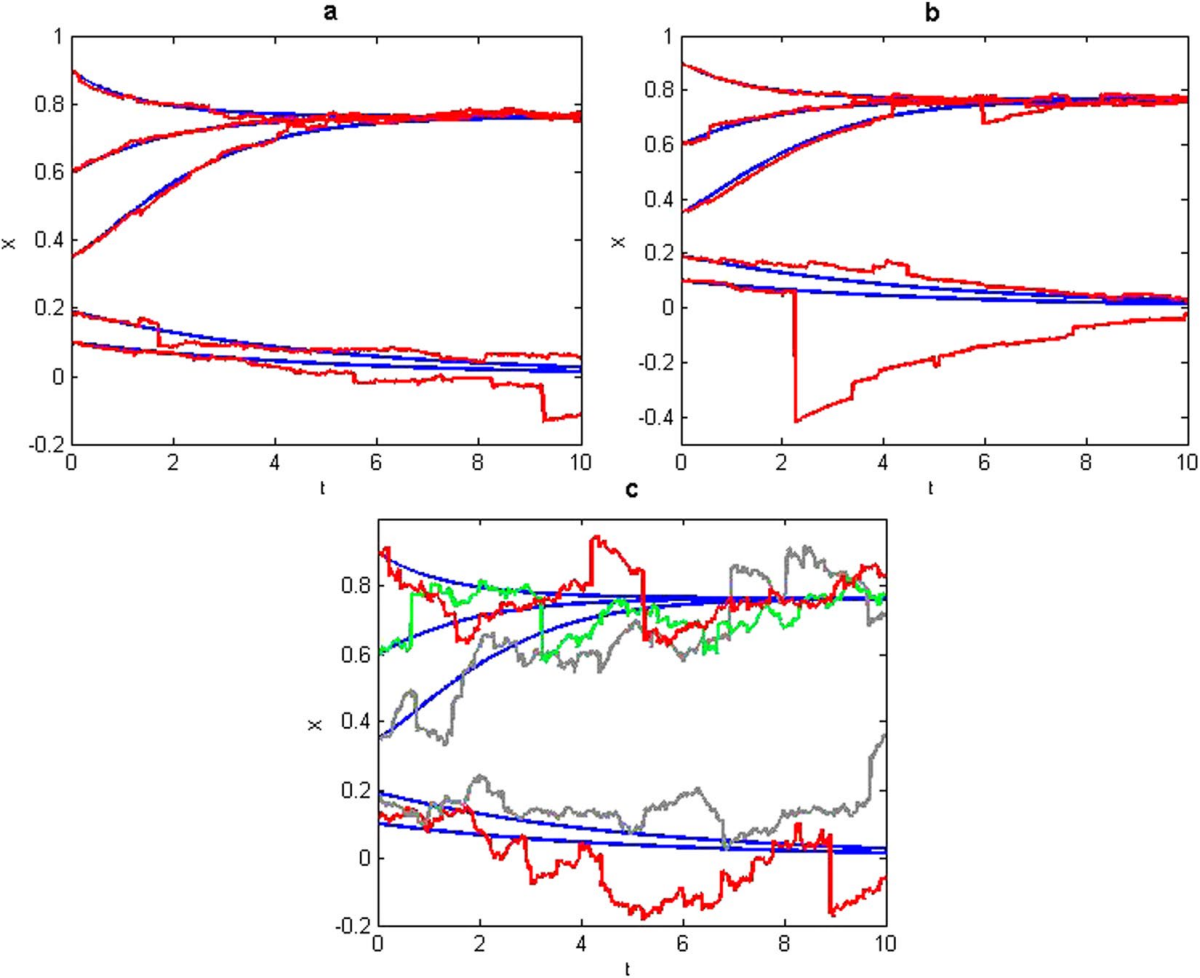
Solutions of deterministic (blue smooth curves) and stochastic mollified (*ε* = 0.08, red, gray and green curves) Amazonian vegetation model with initial conditions *X*_0_ = 0.9, 0.6, 0.35, 0.19, 0.1 when (**a**) *ψ* = 0.01 and *α* = 1.5, (**b**) *ψ* = 0.01 and *α* = 1, (**c**) *ψ* = 0.05 and *α* = 1.5.

According to the existence and uniqueness theorem^12^ (Theorem 7.26 p.202) and under the Lipschitz and growth conditions, the SDE (9) has a unique, adapted, cadlag solution *X*_*t*_. The solutions of the deterministic AV system (7) and the stochastically perturbed system (9) for different initial conditions and values of *α* and *ψ* are shown in Fig. 2.

The generator *A* for SDE (9) is

10 $$Ag(x)={f}^{\varepsilon }(x)g^{\prime} (x)+{\psi }^{\alpha }\,{\int }_{{{\mathbb{R}}}^{1}\backslash \{0\}}\,[g(x+y)-g(x)]{\nu }_{\alpha }(dy)$$

where $$g(x)\in {C}^{2}({{\mathbb{R}}}^{1})$$.

For some years, SDEs with discontinuous drift have attracted substantial attention. Different methods and techniques have been proposed to address the difficulties arising from the discontinuities in the vector field. For instance, in ref. 33 the authors have performed a complete qualitative classification for the isolated singular points, i.e., points with deleted neighbourhoods for which the function (1 + |*b*|)/*δ* ^2^ (where *b*-drift and *σ*-diffusion coefficient) is not locally integrable. This classification allows for the description of the behaviour of solutions in the neighbourhood of isolated singular points and detects the types of points that disturb the uniqueness of solutions. Stochastic bifurcation analysis has also been studied for different types of models: for smooth and discontinuous oscillators^34^, and for piecewise-smooth ODEs in two dimensions with additive Gaussian noise^35^, among others. The dynamical behaviour of stochastically perturbed solutions near the switching manifold has been studied in refs 36 and 37. Attention has also been paid to the solutions^38, 39^, to the transition density function^40^ as well as to the noise-induced regularization^41, 42^ of the SDE with a discontinuous vector field.

## Results and Discussion

### Stability analysis of the metastable forest state

The stochastic AV model (9) exhibits the metastability phenomenon between the two stable states: the savanna *x*_*S*_ and the forest *x*_*F*_. Metastability is defined as a behavioural phenomenon of the solution of the system, which consists of sudden visits with varying durations and frequencies of all domains of attraction^30^. We are particularly interested in performing a stability analysis of the current fertile forest state against stochastic perturbations^2^. To carry out the stability analysis, we study three quantities that provide information on the dynamical behaviour of the system and thus are appropriate for this type of analysis. These are meant to be based on the first exit time, escape probability and stochastic basin of attraction. More information about these quantities can be found in the **Methods** section.

**Figure 3:**
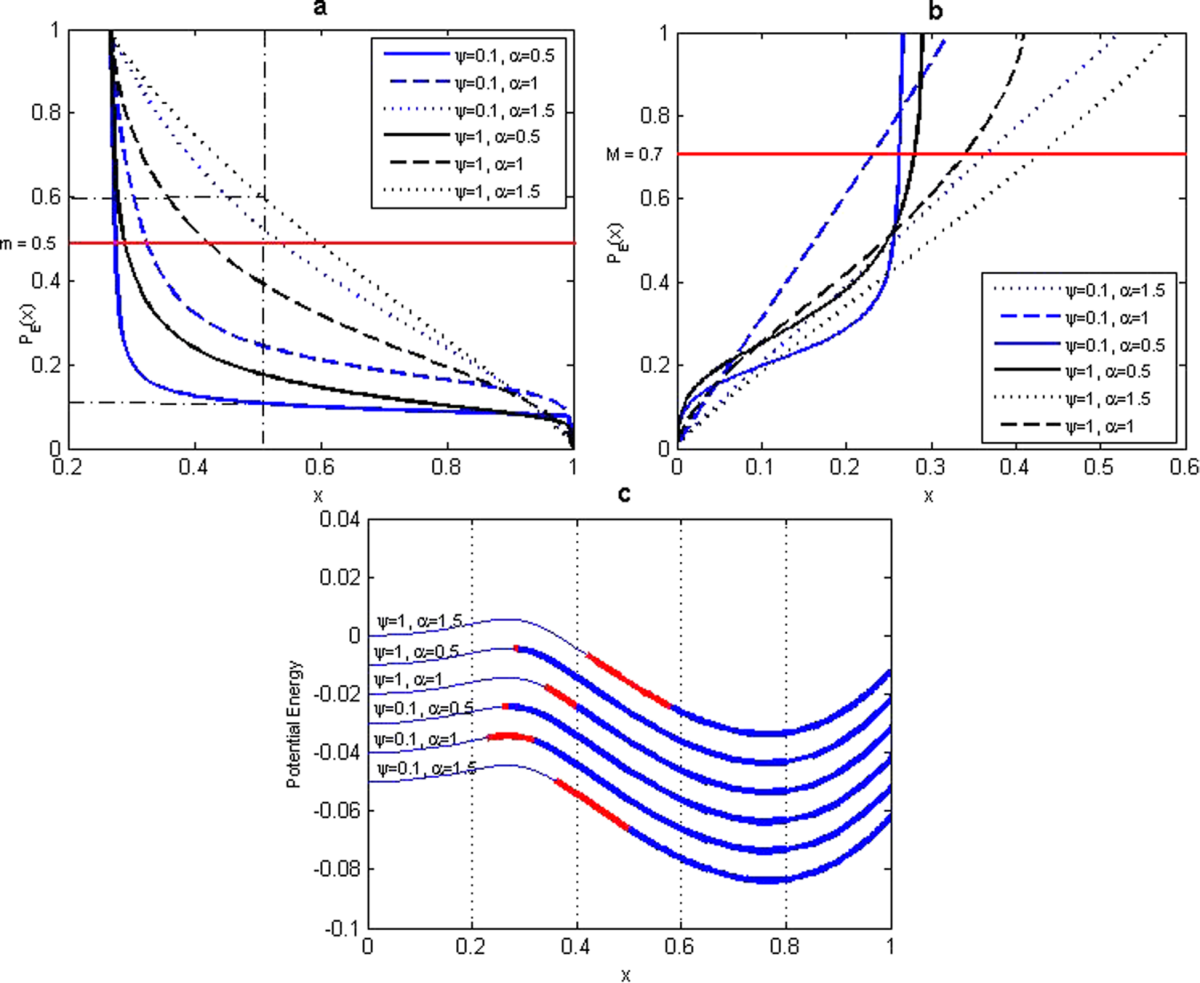
The first definition of SBA for forest state. (**a**) Set *D*_*I*_ defined by escape probability from *D* = (0.2676, +∞) to *D* ^*c*^ = (0, 0.2676). (**b**) Set ${D}_{II}^{c}$ defined by escape probability from ${D}_{I}^{c}=\mathrm{(0},\mathrm{0.52)}$, (0, 0.32), (0, 0.27), (0, 0.29), (0, 0.58), (0, 0.41) to *D*_*I*_ = (0.52, +∞), (0.32, +∞), (0.27, +∞), (0.29, +∞), (0.58, +∞), (0.41, +∞). (**c**) Size and location of the SBA by definition I (*ε* = 0.08) of the fertile forest state. Red part of the well is the set ${D}_{II}^{c}$ and blue thick part is the set *D*_*I*_.

**Figure 4:**
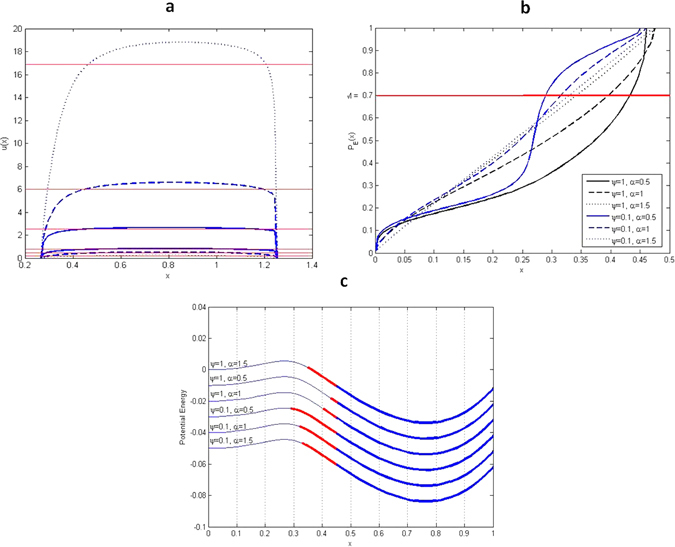
The second definition of SBA for forest state. (**a**) Set *D*_*I*_ defined by mean exit time from *D* = (0.2676, 1.2524). (**b**) Set ${D}_{II}^{c}$ defined by escape probability from ${D}_{I}^{c}=\mathrm{(0},\mathrm{0.46)}$, (0, 0.48), (0, 0.48), (0, 0.45), (0, 0.46), (0, 0.47) to *D*_*I*_ = (0.46, 1.14), (0.48, 1.10), (0.48, 1.07), (0.45, 1.18), (0.46, 1.19), (0.47, 1.21). (**c**) Size and location of the SBA by definition II (*ε* = 0.08) of the fertile forest state. Red part of the well is the set ${D}_{II}^{c}$ and blue thick part is the set *D*_*I*_.

In the following section, we present the main results of stability analysis for the metastable fertile forest state *x*_*F*_ that are based on the Figs 3a–c and 4a–c. By the definitions of the SBA in the case of ${{\mathbb{R}}}^{1}$ the basin consists of the two intervals, i.e. thick blue segment and the thick red segment of the well-potential curve see Figs 3c and 4c. This composition directly relates to the two criteria according to which the basin size is defined^14^. The blue segment is the set of initial conditions ${D}_{I}={\cap }_{i=1}^{n}{D}_{iI}$ whose solutions have a “small“ probability, measured by the level *m* set out in Criterion I, of exit from the neighborhood of the fertile forest attractor. In Fig. 3a, that show the escape probability from domain *D* to *D* ^*c*^, the set *D*_*I*_ described above remains below the red line (*m* = 0.5) that is the probability level established by Criterion I.

Going back to Fig. 3c. the thick red segment is the set of initial conditions ${D}_{II}^{c}={\cup }_{i=1}^{n}{D}_{iII}^{c}$ whose solutions have a “high“ probability, measured by the level *M* set out in Criterion II, of return to the vicinity of fertile forest attractor. In Fig. 3b, that show the escape (return) probability from domain ${D}_{I}^{c}$ to *D*_*I*_, the set ${D}_{II}^{c}$ described above remains over the red line (*M* = 0.7) that is the probability level established by Criterion II. The Fig. 4b,c representing the graphical results of the stability analysis that is based on the second definition of the SBA^43^ have the similar description as Fig. 3b,c. The exception is the Fig. 4a that reproduces the mean exit time from *D*, since in this definition the Criterion I involves mean exit time to define the size of the thick blue segment that is the set of initial conditions *D*_*I*_ whose solutions stay longer *u*(*x*) ≥ *m* in the vicinity of the forests attractor.

### The maximum probability value of the forest-savanna transition is detected under Lévy noise with smaller jumps but with higher frequencies and intensities (*α* = 1.5, *ψ* = 1)

Given the initial condition *x*_0_ = 0.5, the probability that the forest cover, under the noise influence (*α* = 1.5, *ψ* = 1), will undergo the forest-savannah transition is six times higher than the probability of transitions as a result of noise with the parameters *α* = 0.5 and *ψ* = 0.1, see Fig. 3a. This result is a clear indication that small noise jumps strongly contribute to *x*_*F*_ instability.

### The forest cover remains in the fertile forest state longer under Lévy perturbations with smaller jumps of lower intensities (*α* = 1.5, *ψ* = 0.1)

As the intensity of the noise increases, the mean residence time of the forest cover in the *x*_*F*_ basin decreases, thus contributing to the instability of the forest state. The size of the noise jump inversely influences the mean exit time for various levels of noise intensity: for high noise intensity (say *ψ* = 1) with the increase of the jump size, which raises the exit time, an enhancement of the forest state stability is seen; however, for a low noise intensity (say *ψ* = 0.1) with the increase of the jump size, which reduces the exit time, the forest state becomes more unstable, see Fig. 4a.

### The fertile forest state is the largest stability basin under symmetric Lévy process with large jumps of low intensity (*α* = 0.5, *α* = 1 and *ψ* = 0.1

The overall stability results obtained from the two basins’ definitions are similar. However, these two definitions provide additional information, which is complementary and provides a complete view of the state stability. In the case of the first SBA definition, which is based on the escape probability, the strongest contribution to the size of the basin is due to the set of initial points (0.27, +∞) (resp. (0.32, +∞)) for *α* = 0.5 (resp. *α* = 1) whose solutions have the decreased escape probability from the deterministic basin of attraction. However, the entrance probabilities do not significantly contribute to the size of the SBA (see Fig. 3a–c). The smaller basins, i.e. (0.36, +∞) and (0.42, +∞), are obtained for noise with smaller jumps (*α* = 1, 5) independently of the intensity, and therefore are the cause of the strongest state instability.

In the case of the second SBA definition, on the basis of the mean exit time and escape probability, the time has the same contribution (due to the choice of the criterion *m* = *AMET* average mean exit time) to the length of the basin for the different noise parameters *α* and *ψ*. What differentiates the length of the basins is the second criterion based on the escape probability, see Fig. 4a–c. Consequently from the second SBA definition, the shorter basins (0.40, +∞) (resp. (0.43, +∞)) are obtained for noise with higher intensity and larger jumps *α* = 1, *ψ* = 1 (resp. *α* = 0.5, *ψ* = 1).

**Figure 5:**
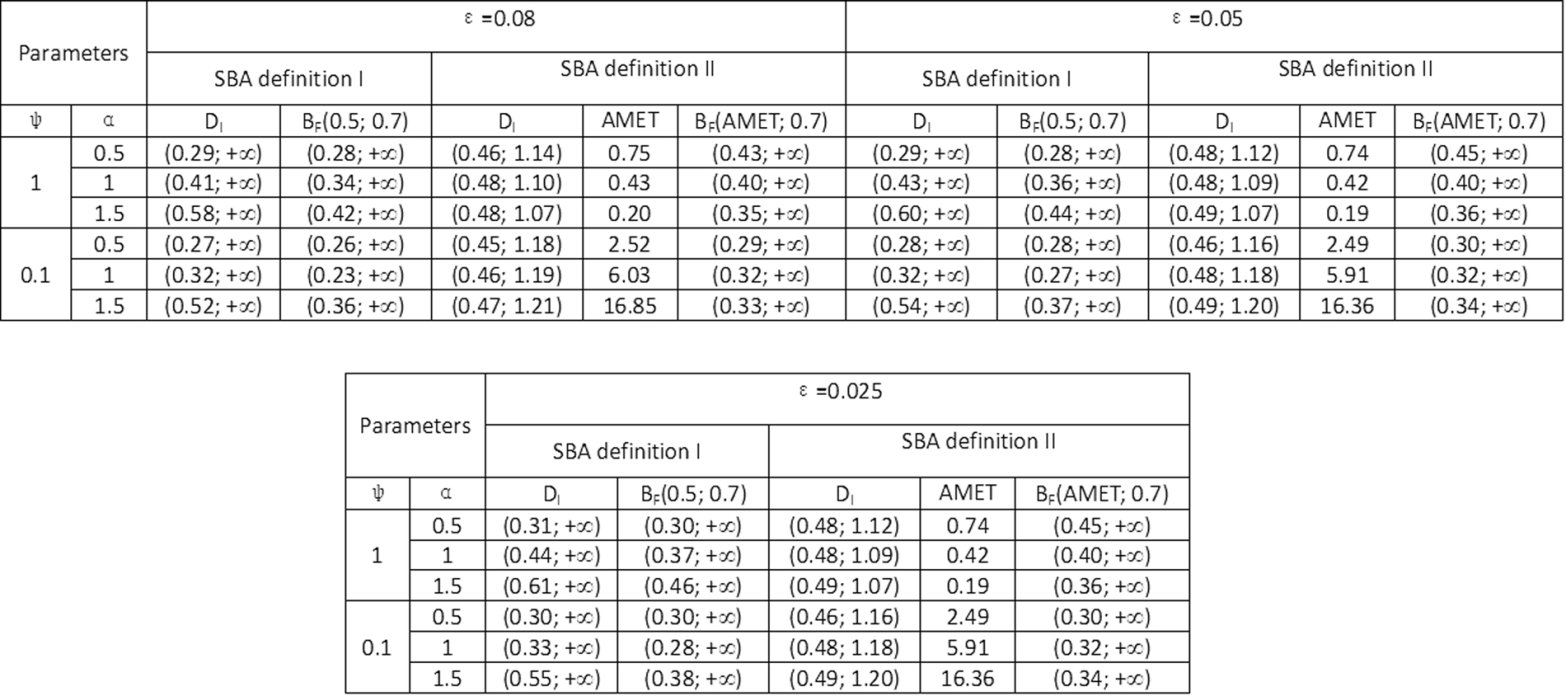
The SBA of the fertile forest state in the mollified Amazonian Vegetation Model with *ε* = 0.08, 0.05, 0.025.

### Even very small threats to the forest state stability represents Lévy noise with large jumps of low intensity (*α* = 0.5, *α* = 1 and *ψ* = 0.1)

Lévy noise with small jumps (*α* = 1.5) as well as noise with high intensity (*ψ* = 1) significantly accelerates the transition between the forest and savanna states, thus causing high instability of the forest. The size of the SBA of *x*_*F*_ does not undergo significant variations with the decrease in the values of the mollification parameter *ε* from the threshold *ε* = 0.08. This conclusion is confirmed by the results in Figure 5, which contains the SBA of the fertile forest state in the mollified Amazonian vegetation model with different values for the parameter *ε* = 0.08, 0.05 and 0.025.

We can explain our results from an ecological point of view, associating Lévy noise with large jumps of low intensity (*α* = 0.5, 1 and *ψ* = 0.1) to low-intensity fires that occur in non-drought years. However, during years of severe drought and high-intensity fires, the Lévy noise with small jumps of high intensity (*α* = 1.5 and *ψ* = 1) significantly accelerates the transition between the forest and savanna states, thereby causing height instability of the forest.

## Conclusion

For the tipping elements in the Earth’s climate system the most important issue to address is how stable the desirable state is against random, possibly large perturbations. Thus, we performed a stability analysis of the metastable fertile forest state in a stochastically perturbed Amazonian vegetation model with a discontinuous right-hand side.

Considering that the usual notion of solutions is not suitable for a piecewise-smooth dynamics for our research, we used the generalized definition of the solution from Filippov theory. The AV deterministic system does not define the vector field *f*(*t*, *x*(*t*)) if *x*(*t*) is on a switching boundary (tipping point *x* = *X*_*crit*_). To overcome this problem, we extend a discontinuous system into a Fillipov convex differential inclusion. The solution concept, in the sense of Filippov theory, guarantees the existence of a solution for this CDI by the assumption that the set-valued function *F*(*t*, *x*) is upper semi-continuous. The type of vector field, Amazonian vegetation CDI, surrounds the switching boundary *x* = *X*_*crit*_ and is called a repulsive (repelling) sliding mode, for which the uniqueness of solutions is not guaranteed. In fact, the IVP with the initial condition *x*_0_ = *X*_*crit*_ has three possible solutions. To overcome the problem of non-unique solutions in the AV non-smooth dynamical system, and to be able to apply stability analysis techniques for the metastable states in the case of stochastically perturbed dynamical systems, we have considered a smooth approximation of the discontinuous vector field. The smooth approximation was been performed by mollification techniques, and we have used the convolution kernel generated by Gaussian function as the mollifier. The mollification of the original AV model changes the dynamical behavior of the system in the neighborhood of the discontinuity *x* = *X*_*crit*_ = 0.3, transforming the repulsive sliding mode into the unstable equilibria *x*_*A*_ = 0.27 (resp. 0.28, 0.29) for *ε* = 0.08 (resp. 0.05, 0.025).

Research in the environmental sciences has provided empirical evidence that tree cover experiences drastic variations or sharp transitions in response to climate changes and other stressors. Recently, to model these abrupt pulses, burst-like or extreme events have been given higher priority to Lévy perturbations with jumps, because their properties, such as heavy-tailed distributions and stochastically continuous sample paths, provide the greatest precision to fit the described phenomena. Therefore, in our stochastic AV model, we have considered perturbations of symmetric *α*-stable Lévy type, which under the considered conditions, exhibit metastability between the two stable states: savanna *x*_*S*_ and forest *x*_*F*_. We have been particularly interested in performing a stability analysis of the current fertile forest state against stochastic perturbations. To perform the stability analysis, we have calculated the following three quantities: mean first exit time, escape probability and stochastic basin of attraction, which provide information about the dynamical behavior of the system and thus are appropriate for this type of analysis.

Our main conclusions include the following three points: the maximum probability value of the forest-savanna transition is detected under Lévy motion with small jumps of high frequency and intensity (*α* = 1.5, *ψ* = 1), the forest cover remains in the fertile forest state longer under Lévy perturbations with small jumps of low intensity (*α* = 1.5, *ψ* = 0.1); and the fertile forest state is the largest stability basin under the symmetric Lévy noise with the large jumps of low intensity *α* = 0.5, *ψ* = 0.1. The results of our analysis also show that even a small threat to forest state stability represents Lévy noise with large jumps of low intensity (*α* = 0.5, *ψ* = 0.1). In contrast, a Lévy noise with smaller jumps (*α* = 1.5) as well as noise with higher intensity (*ψ* = 1) significantly accelerate the transition between the forest and savanna states, thereby causing high instability of the forest.

## Methods

### Condition for local uniqueness of the solution of the differential inclusion

The solution of the differential inclusion (3) with *x*_0_ ∈ Σ is locally unique in forward time if

(i) The projections of the vector field point to the same side of Σ, i.e. the solution exposing a transversal intersection to the switching boundary:
  11 $$n{({x}_{0})}^{T}{f}_{-}({t}_{0},{x}_{0})\cdot n{({x}_{0})}^{T}{f}_{+}({t}_{0},{x}_{0}) > \mathrm{0,}\,\forall n(x)\in \partial h(x),$$
  or if
(ii) The projections point to Σ, i.e. the solution being an attractive sliding mode:

12 $$n{({x}_{0})}^{T}{f}_{-}({t}_{0},{x}_{0}) > 0\,{\rm{and}}\,n{({x}_{0})}^{T}{f}_{+}({t}_{0},{x}_{0}) < \mathrm{0,}\,\forall n(x)\in \partial h(x),$$

where the normal *n*(*x*) perpendicular to a locally smooth switching boundary Σ is given by the gradient of *h*(*x*):

13 $$n(x)=\nabla h(x),$$

or if *h*(*x*) is non-smooth, by using the generalized differential of *h*(*x*) (for more detail see ref. 20, section 2.3):

14 $$n(x)=\partial h(x),$$

where ∂*h*(*x*) is assumed to be bounded.

### Mollification method

The general idea of the mollification method is to convolve (i.e. a mathematical operation on two functions that is defined as the integral of the product of these functions after one is reversed and shifted) a discontinuous function with a mollifier (i.e. a smooth function with special properties) to get the piecewise-smooth continuous function, that still remains close to the original generalized function.

Mollifier were proposed by Friedrichs^44^ in the study of partial differential equations and is defined as follows:

A real function *η*_*ε*_ is called a Friedrichs’ mollifier if

15 $${\eta }_{\varepsilon }(x)=\frac{1}{\varepsilon }\eta (\frac{x}{\varepsilon }),\,\eta \in {C}_{0}^{\infty }({\mathbb{R}}),\,x\in {\mathbb{R}},$$

with the generator *η* satisfying the following conditions:

(i) *η*(*x*) ≥ 0, $$x\in {\mathbb{R}}$$;
(ii) *η*(*x*) = 0, if |*x*| > 1;
(iii) $${\int }_{{\mathbb{R}}}\,\eta (x)dx=1$$;

Among the possible choices for the generator *η* we use the Gaussian function $\eta (x)=\frac{1}{\sqrt{\pi }}\,\exp \,(-{x}^{2})$. However, the convolution kernel ${\eta }_{\varepsilon }=\frac{1}{\varepsilon \sqrt{\pi }}\,\exp \,(-\tfrac{{x}^{2}}{{\varepsilon }^{2}})$ generated by the Gaussian function does not have a compact support. Thus, to be able to use the Gaussian kernel as the mollifier, instead of a compact support, it is required that the generator’s moments *μ*_*k*_ must be finite^28^, i.e.,

16 $${\mu }_{k}={\int }_{{\mathbb{R}}}\,{x}^{k}\eta (x)dx\in {\mathbb{R}},\,\,\,\,{\textstyle \text{for}}\,{\textstyle \text{all}}\,{\textstyle \text{integers}}\,k.$$

The convolution operator as well as the mollification are defined as ref. 45:

If $f:U\to {\mathbb{R}}$ is locally integrable, then its mollification is represented by

17 $${f}^{\varepsilon }={\eta }_{\varepsilon }\ast f\,\,\,\,{\rm{i}}{\rm{n}}\,\,{U}_{\varepsilon },$$

that is,

18 $${f}^{\varepsilon }(x)={\int }_{U}\,{\eta }_{\varepsilon }(x-y)f(y)dy={\int }_{B(0,\varepsilon )}\,{\eta }_{\varepsilon }(y)f(x-y)dy\,\,\,\,{\rm{f}}{\rm{o}}{\rm{r}}\,x\in {U}_{\varepsilon },$$

where *B*(0,*ε*) is the closed ball with the center 0 and radius *ε* > 0, $U\subset {{\mathbb{R}}}^{n}$ is open with the boundary ∂*U* and

19 $${U}_{\varepsilon }=\{x\in U\,|{\rm{d}}{\rm{i}}{\rm{s}}{\rm{t}}\,(x,{\rm{\partial }}U) > \varepsilon \}.$$

The mollified function *f* ^*ε*^(*x*) defined in this way possesses the desired properties (proof can be seen in ref. 45):

(i) *f* ^*ε*^ ∈ *C* ^∞^(*U*_*ε*_). The mollified function becomes infinitely differentiable;
(ii) *f* ^*ε*^ → *f* a.e. as *ε* → 0. The mollified function almost everywhere converges to the original one as the parameter *ε* shrinks;
(iii) If *f* ∈ *C*(*U*), then *f* ^*ε*^ → *f* uniformly on the compact subset of *U*;
(iv) If 1 ≤ *p* < ∞ and $f\in {L}_{loc}^{p}(U)$, then *f* ^*ε*^ → *f* in ${L}_{loc}^{p}(U)$.

### Lévy perturbations

A brief summary of the main properties of Lévy type stochastic process is useful for the understanding of the analysis method and evaluation of the results. The stochastic process that models the behavior of the landscape variable tree cover has to be a positive process. Thus, we ponder the following fore cases.

i) The **First case** is the *Lévy process absorbed at level* 0. Let (*X*, *P*_*x*_) be a initial stable Lévy process starting at *x* > 0 and *T* = inf{*t* ≥ 0: *X*_*t*_ ≤ 0} is the first hitting time at negative half-line. The probability measure ${{\mathbb{P}}}_{x}$ is the law under *P*_*x*_ of the process $(X,{{\mathbb{P}}}_{x})$ defined as
  20 $${X}_{t}{{\bf{1}}}_{\{t < T\}},\,t\ge 0.$$
  and it is called killed process or absorbed at level 0. If initial Lévy process (*X*, *P*_*x*_) has negative jumps it crosses the level 0 by jumping^46^, so $(X,{{\mathbb{P}}}_{x})$ vanishes with a jump at 0, i.e. the lifetime of the process is almost surely finite.
ii) If (*X*, *P*_*x*_) has no negative jumps then $(X,{{\mathbb{P}}}_{x})$ *vanishes continuously at* 0, belonging to the **Second case** of the positive (${{\mathbb{R}}}_{+}$-valued) processes.
iii) The **Third case** is the *Lévy process conditioned to stay positive*. The following construction of the law ${{\mathbb{P}}}^{\ast }$ is one of the possible constructions that justifies considering $(X,{{\mathbb{P}}}^{\ast })$ as the Lévy process (*X*, *P*_*x*_) conditioned to stay positive:
  21 $${{\mathbb{P}}}_{x}^{\ast }(A)=\mathop{\mathrm{lim}}\limits_{t\to \infty }\,{P}_{x}(A|T > t),\,x > 0,\,t\ge \mathrm{0,}\,A\in { {\mathcal F} }_{t},$$
  where ${ {\mathcal F} }_{t}$ is the Borel filtration generated by *X*, i.e. ${ {\mathcal F} }_{t}=\delta ({X}_{s},s\le t)$. The infinitesimal generators for these processes were explicitly computed in ref. 46. These three cases of processes can be considered for the AV model in the case of the perturbations such as the pests, diseases^8^, deforestation^2^, etc. For which, in most of cases, the level *X* = 0 represents absorbing state.
iv) The **Fourth case** is the *symmetric α*-*stable Lévy process*. A symmetric *α*-stable scalar Lévy motion ${L}_{t}^{\alpha }$ with 0 < *α* < 2 is defined in a similar way as a Brownian motion, excepting two following properties: i) stationary increments ${L}_{t}^{\alpha }-{L}_{s}^{\alpha }$ and ${L}_{t-s}^{\alpha }$ have the same symmetric *α*-stable distribution, i.e. ${S}_{\alpha }((t-s{)}^{\tfrac{1}{\alpha }}\mathrm{,0,0)}$ and ii) stochastically continuous sample paths, i.e., for every *s* > 0, ${L}_{t}^{\alpha }\to {L}_{s}^{\alpha }$ in probability, as *t* → *s*.

The probability density function for ${L}_{t}^{\alpha }$ is defined by

22 $${t}^{-\tfrac{1}{\alpha }}{f}_{\alpha }({t}^{-\tfrac{1}{\alpha }}x),$$

where *f*_*α*_ is the probability density function for the standard symmetric *α*-stable random variable $X\sim {S}_{\alpha }(1,0,0)$ (for more details see ref. 12).

The generating triplet of ${L}_{t}^{\alpha }$ is (0, 0, *ν*_*α*_), with the jump measure, i.e. the expected value of the number of jumps of size *dy* during the unit time, defined as:

23 $${\nu }_{\alpha }(dy)={c}_{\alpha }\frac{dy}{{|y|}^{1+\alpha }},\,\alpha \in (0,2),$$

where *c*_*α*_ is the intensity constant. The jump measure controls the intensity and size of the jumps of the process. So the *α*-stable Lévy process has finite variation as well as larger jumps with lower jump frequencies for small values of *α* (0 < *α* < 1) while it has unbounded variation as well as smaller jumps with higher jump probabilities when *α* ∈ [1, 2).

### Mean first exit time

It is defined as the first exit time from a deterministic domain $D\subset {{\mathbb{R}}}^{1}$ of attraction of *x*_*F*_ as follows:

24 $$\tau (\omega ,x)=inf\,\{t\ge 0,\,\,{X}_{t}(\omega ,x)\notin D\},$$

and the mean exit time or the mean residence time of the process in the forest domain is denoted as $u(x)\triangleq {\mathbb{E}}\tau (\omega ,x)\ge 0$. It has been proven^12^ that the mean exit time of the stochastic system (9) for an orbit starting at *x* ∈ *D*, satisfies the following nonlocal partial differential equation with an external boundary condition

25 $$\begin{array}{l}Au(x)=-1,\\ \,\,\,u(x)=0,\,x\in {D}^{c},\end{array}$$

where *A* is the generator defined in (10) which can be interpreted as $Au=\mathop{\mathrm{lim}}\limits_{t\to 0}\,\frac{{\mathbb{E}}u({x}_{t})-u}{t}$, for every $u\in {C}^{2}({{\mathbb{R}}}^{1})$.

### Escape probability

The likelihood that the tree cover process *X*_*t*_ exits firstly from the forest domain of attraction *D* by landing in the set *U* ∈ *D* ^*c*^ belonging to the savanna domain is represented by

26 $$p(x)={\mathbb{P}}\{{X}_{\tau }(x)\in U\}$$

and solves the following differential-integral equation with Balayage-Dirichlet boundary condition

27 $$\begin{array}{ccc}Ap(x) & = & 0,\quad x\in D,\\ p(x) & = & \{\begin{array}{cc}1, & x\in U,\\ 0, & x\in {D}^{c}\backslash U.\end{array}\end{array}$$

### Stochastic basin of attraction

The third quantity that we will use is the stochastic basin of attraction, introduced in refs 14 and 43. The SBA is an important geometric structure that helps to perceive and describe the metastable behavior of a system. It is crucial to have an approach for describing the basin of attraction and quantifying its shape and size for theoretical and practical reasons^14^.

By **Definition I**: SBA of the attractor *K* with the open deterministic domain of attraction *D* is the set ${B}_{K}(m,M)=[{\cup }_{i=1}^{n}{D}_{iII}^{c}]\cup [{\cap }_{i=1}^{n}{D}_{iI}]$, where *D*_*iI*_ = {*x* ∈ *D* | *p*_*i*_(*x*) < *m*}, ${D}_{iII}^{c}=\{x\in {D}_{iI}^{c}\,|\,{p}_{i}(x) > M\}$, *D*_*i*_ are the domains of attraction of nearby attractors *K*_*i*_ and *p*(*x*) is the escape probability defined in (26).

By **Definition II**: SBA of the attractor *K* with the open deterministic domain of attraction *D* is the set ${B}_{K}(m,M)\triangleq {D}_{I}\cup {D}_{II}^{c}$, where *D*_*I*_ = {*x* ∈ *D* | *u*(*x*) ≥ *m*}, ${D}_{II}^{c}=\{x\in {D}_{I}^{c}\,|\,p(x)\ge M\}$, *u*(*x*) is the mean first exit time defined in (24) and *p*(*x*) is the escape probability defined in (26).
